# Advancing Versatile Ferroelectric Materials Toward Biomedical Applications

**DOI:** 10.1002/advs.202003074

**Published:** 2020-12-03

**Authors:** Wenjun Wang, Jianhua Li, Hong Liu, Shaohua Ge

**Affiliations:** ^1^ Department of Biomaterials, School and Hospital of Stomatology, Cheeloo College of Medicine Shandong University & Shandong Key Laboratory of Oral Tissue Regeneration & Shandong Engineering Laboratory for Dental Materials and Oral Tissue Regeneration Jinan 250012 China; ^2^ State Key Laboratory of Crystal Materials Shandong University Jinan 250013 China

**Keywords:** biomedicine, ferroelectric materials, physical stimuli

## Abstract

Ferroelectric materials (FEMs), possessing piezoelectric, pyroelectric, inverse piezoelectric, nonlinear optic, ferroelectric‐photovoltaic, and many other properties, are attracting increasing attention in the field of biomedicine in recent years. Because of their versatile ability of interacting with force, heat, electricity, and light to generate electrical, mechanical, and optical signals, FEMs are demonstrating their unique advantages for biosensing, acoustics tweezer, bioimaging, therapeutics, tissue engineering, as well as stimulating biological functions. This review summarizes the current‐available FEMs and their state‐of‐the‐art fabrication techniques, as well as provides an overview of FEMs‐based applications in the field of biomedicine. Challenges and prospects for future development of FEMs for biomedical applications are also outlined.

## Graphical abstract

Ferroelectric materials (FEMs), which are able to sense and convert mechanical, thermal, electrical, and optical energy, possess multiple capabilities to interface with various biological processes, thus demonstrating great potential to work for biomedicine.

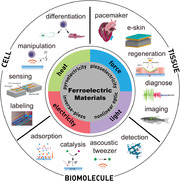

## 1 Introduction

Human bodies can sense and respond to different physical cues from the surrounding environment and perform physiological activities correspondingly. Over the past years considerable efforts have been made to utilize physical stimuli to understand and manipulate biological processes. For instance, in terms of electricity, the rapid development of bioelectronics has created many successful biomedical devices including blood pressure sensors, deep‐brain stimulator, and cardiac pacemakers.^[^ ^1^ ^]^ In terms of light, the cutting‐edge imaging technologies integrated with optical nanomaterials allow us to study biological science at molecular scale.^[^ ^2^ ^]^ In addition, the combination of optics and genetics, that is, optogenetics, has enable the activation and regulation of neuronal functions and the exploration of brain science.^[^ ^3^ ^]^ As regards mechanical stimuli, forces from the extracellular matrix (ECM) can be used to regulate cell behavior via mechanisms such as mechanotransduction.^[^ ^4^ ^]^ It should be recognized that materials are often needed to deliver those physical cues to the biological system, hence, materials with energy‐transducing ability are of great importance to both biological sensing and manipulation.

FEMs were discovered in 1920 when J. Valasek first demonstrated the spontaneous polarization of Rochelle salt could be macroscopically inverted by the applied external electric field.^[^ ^5^ ^]^ Since then FEMs have attracted extensive attention due to their excellent properties such as ferroelectricity, pyroelectricity, piezoelectricity, inverse piezoelectricity, and nonlinear optics. As FEMs can be utilized for a broad range of stimuli and convert one physical stimuli into another form of stimuli, they serve as versatile transducers facilitating their broad applications in sensing, actuation, data storage, energy harvesting, electro‐optic devices etc.^[^ ^6^ ^]^ Until very recently, we have witnessed exciting progress in the development of FEMs with their multifunctional properties in the field of biomedicine, while many naturally‐derived and artificial FEMs have been synthesized or manufactured. One typical example is that the manmade ferroelectric lead zirconate titanate (Pb(Zr*_x_*Ti_1−_ *_x_*)O_3_, PZT) with a high piezoelectric coefficient has been extensively employed for ultrasound transducer, which has been broadly applied as a diagnostic tool for medical visualization and ultrasound‐guided therapies.^[^ ^7^ ^]^

In this review, fundamentals of FEMs and their interactions with light, heat, force, and electrical field will be briefly summarized first. Currently available together with newly developed FEMs will then be presented, followed by some latest fabrication techniques being overviewed. We will then emphasize discussing the biological responses to the FEM‐mediated electrical and mechanical stimuli at molecular, cellular, and tissue levels, and how FEMs in turn sensing biological activities, as well as highlight their implications and potential applications. FEMs‐based nonlinear optics for bioimaging and light‐mediated therapy will be discussed in the end. This review is addressed to a converging audience, interested in the latest developments of FEMs with prospective use in biomedicine.

## 2 Working Mechanisms of FEMs

**Figure 1:**
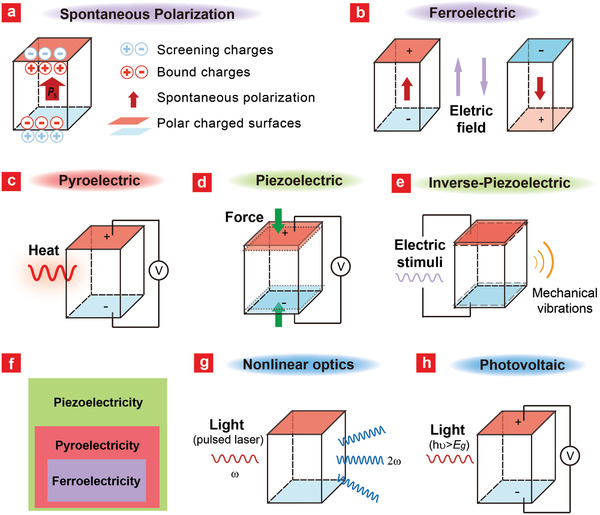
Working mechanisms of FEMs interacting with physical stimuli. Diagrams illustrating the corresponding mechanisms in a unit cell: a) spontaneous polarization (*P*_s_) with induced polar charged surfaces; b) ferroelectricity, inversion of polarization by applied electric field; c) pyroelectricity, generation of electric signal upon temperature change; d) piezoelectricity, generation of electric signal by applied force; e) inverse‐ piezoelectricity, generation of mechanical strain/vibrations by applied electric signal. f) The relationship between piezoelectricity, pyroelectricity, and ferroelectricity. g) Nonlinear optics, generation of harmonic photon when excited by a pulsed laser; h) Photovoltaic effect, generation of photovoltage when excited by illumination with photon energy (*hν*) higher than the band gap (*E*_g_).

In order to explain the specific properties of FEMs for biomedical applications, it is necessary to clarify some fundamental concepts and mechanisms of FEMs and their related properties. The characteristics of ferroelectrics is defined as the possession of spontaneous polarization (*P*_s_), as shown in **Figure** **1**a, that can be inverted by an external electric field (*E*) (Figure 1b), resulting in a *P*_s_–*E* hysteresis loop. The existence of *P*_s_ results in the formation of positive and negative bound polarization charge at the corresponding polarized surfaces.^[^ ^8^ ^]^ In ambient conditions, the bound surface charges are mainly compensated by external screening via surface charges, including injected charges, internal charges from defects, or surface relaxation, and in most cases the adsorbed charged species from ambient environment such as hydroxyl ions, hydrocarbons, and protons.^[^ ^9^ ^]^ For instance, the positive polarization charge will be screened by anions and negatively‐charged molecules when polarized FEMs are immersed in an aqueous solution.

For most FEMs, the *P*_s_ only exists over a certain range of temperatures. Within this range, the value of *P*_s_ varies and charge generation occurs on the surfaces when the FEMs undergo a temperature change, which is defined as pyroelectricity (Figure 1c). Pyroelectricity has been observed in many biological systems as well, and it is believed to be of fundamental physiological importance to biological function such as sensory organs.^[^ ^10^ ^]^ If the temperature is raised to a certain point (Curie temperature, *T*_c_), a ferroelectric‐to‐paraelectric phase transition occurs where *P*_s_ drops to zero. Therefore, when the *P*_s_ decreases with increasing temperature (*T*), the generated surface charges will form a potential difference across the polar axis and from which the pyroelectric current can be measured. The output current *i* can be expressed as *i*  =  *pA* d*T*/d*t*, where *A* is the area of surface electrode, *p* (µC  m^−2^ K^−1^) is the pyroelectric coefficient, which can be further defined as *p* = d*P*_s_/d*T*.^[^ ^11^ ^]^

Piezoelectricity refers to the generation of electric charges of equal magnitude and with opposite symbols on the ferroelectric surfaces when they are subjected to directional mechanical stress, thus the induced electrical field is parallel to the stress direction (Figure 1d). Piezoelectricity is well known to be a fundamental property in many biological tissues like bone, tendon, teeth, muscles, and nerves.^[^ ^12^ ^]^ For instance, the electric response of bone to the applied mechanical stimuli is proven to play an important role in bone growth and remodeling.^[^ ^13^ ^]^ Conversely, inverse piezoelectricity means the generation of a mechanical strain in response to an applied electrica field (Figure 1e). Basically, the piezoelectric coupling of mechanical and electrical behaviors for a piezoelectric crystal can be depicted as *S_ij_* = *d_kij_E_k_* (*i*, *j*, *k* = 1, 2, and 3), where *S*, *d*, and *E* donate the second‐rank strain tensor, piezoelectric coefficient, and the electric field, respectively; *i*, *j*, and *k* represent *x*, *y*, and *z* of the Cartesian reference frame.^[^ ^14^ ^]^ In a classic plate capacitor model, the piezoelectric performance as a function of the strain rate can be expressed by *i* = *d*_33_ *EA*(d*ε*/d*t*), where *i* is the output current, *d*_33_ is the piezoelectric coefficient, *E* is Young's modulus, *A* is the cross‐sectional area, *ε* is the applied strain, and *t* is the time.

Given the relationship of piezo‐, pyro‐, and ferroelectricity shown in Figure 1f, it is logical to speculate that ferroelectricity has important physiological functions for biological systems as well, although the existence of such “bioferroelectricity” has not been firmly established.^[^ ^15^ ^]^ It is expected that numerous biological systems possess the basic elements of ferroelectricity; that is, polarized states and coercive fields needed to reorient dipoles in space. Some studies suggested the possible importance of ferroelectricity in cellular processes or its influence on biomolecules.^[^ ^16^ ^]^ For example, Leuchtag et al. first proposed the ferroelectric approach to the analysis of the conformation transition in voltage‐gated ion channels and until very recently Li et al. have demonstrated the first macroscopic observation of ferroelectric switching in a biological elastin system.^[^ ^15^ ^]^ Therefore, there is huge unknown field to be explored for the basic understanding of biological ferroelectricity.

Besides the above working mechanisms (pyro/piezo/ferroelectricity) of FEMs that interact with heat, force, and electric field, respectively, it has also been shown that the coupling of light with FEMs present unique nonlinear optical effect, photovoltaic, acousto‐optic, and electro‐optic properties.^[^ ^17^ ^]^ Nonlinear optics of FEMs refer to the ability to efficiently produce multi‐directional second or third harmonic generation (SHG or THG) for a variety of geometries in a broad spectral range. The process of SHG (Figure 1g), for instance, is a second‐order nonlinear optical process, where two photons at the frequency (*ω*) interact with certain asymmetric materials, combine, and produce a new single photon with twice the frequency (2*ω*) of the two incident photons.^[^ ^18^ ^]^ The optical response of such nonlinear media can be specified by means of the induced polarization *P*(*ω*) (dipole moment per unit volume) as a power series of the amplitude *E*(*w*) of the electric field of the incident light: *P*(*ω*) = *χ* ^(1)^ *E*(*ω*) + *χ* ^(2)^ *E*(*ω*)^2^ + *χ* ^(3)^ *E*(*ω*)^3^ +…, where the coefficient *χ* ^(^ *^n^* ^)^ is the *n*th‐order susceptibility of the material. In nonlinear optical microscopy, SHG depends on two photons striking a site at once, therefore SHG employs pulsed lasers in the near infrared wavelength range, which could reduce nonspecific phototoxicity as well as enhance photo penetration.^[^ ^19^ ^]^

In addition, when under illumination with photon energy higher than the band gap (*hν* > *E*_g_) of the FEMs, they are able to generate electron–hole pairs that are separated spontaneously by the built‐in electric field induced by the intrinsic polarization, resulting in a photocurrent as well as an above‐bandgap photovoltage (Figure 1h).^[^ ^20^ ^]^ This polarization‐induced charge‐separation mechanism (i.e., ferroelectric–photovoltaic effect) is fundamentally different with conventional photovoltaic theory in semiconductor.^[^ ^21^ ^]^ It has been shown that the photovoltaic performance of ferroelectrics is highly associated with their polarization strength and domain walls.^[^ ^22^ ^]^ Conventional FEMs like ferroelectric oxides can only harvest ultraviolet light in the solar spectrum due to their wide band gaps (*E*_g_ at 2.7–4 eV). Considerable effort has been devoted to engineer the ferroelectric bandgaps to match a broader spectrum region via chemical substitution and ordering, such as introduction of transition‐metal atoms into the host lattices, to expand their utilization of the solar energy.^[^ ^23^ ^]^ This could be beneficial to biomedical purpose as well, because at longer wavelengths (700–1000 nm) ferroelectric–photovoltaic effect could be able to work in the transmission window of biological tissue. Therefore, photo‐activated FEMs might offer unique opportunities to explore light energy conversion toward potential applications for bioimaging and bioelectronics.

## 3 Current FEMs and Their Recent Fabrication Techniques

The above versatile physical properties of FEMs are derived from their unique crystal structures and compositions. Materials with piezoelectricity must possess a noncentrosymmetric crystal structure. Pyroelectricity requires that crystal structures be noncentrosymmetric, meanwhile, possess a unique polar axis. Among thirty‐two classes of crystal structures, only twenty crystal classes demonstrate piezoelectricity. In addition, ten of these twenty classes have spontaneous polarization within the structure, which render them pyroelectric properties. Crystal structures of ferroelectrics are a subgroup of those of pyroelectrics, whose spontaneous polarization within the structure can be inversed by an applied external electric field with enough magnitude. Taken together, FEMs belong to a larger group of pyroelectric materials that possess a unique polar axis, which belongs to a larger group of piezoelectric materials.

The compositions of ferroelectrics in the past decades has been dominated by inorganic materials such as barium titanate (BaTiO_3,_ BTO), and organic polymers such as polyvinylidene fluoride (PVDF). BTO along with alkaline niobate (e.g., KNbO_3_, LiNbO_3_), and PZT, belong to the ABO_3_ perovskite‐type family, which are the oxide‐based FEMs with excellence performance that have attracted the most attention in the field of sensors, memories, and optical devices.^[^ ^17c^ ^]^ However, in the rapid developing biomedical fields including wearable devices, biosensors, and implants, the requirements for practical materials to be lightweight, mechanically flexible, solution processible, biocompatible, and even biodegradable,^[^ ^24^ ^]^ motivate the exploration of organic compounds, liquid crystals, and organic‐inorganic hybrids for FEMs.^[^ ^25^ ^]^ In addition, some native proteins (e.g., elastin^[^ ^15^, ^26^ ^]^), self‐assembled peptide nanoparticles,^[^ ^27^ ^]^ and inorganic bio‐substances (e.g., hydroxyapatite^[^ ^28^ ^]^) with ferroelectric features have been discovered recently. Though the performance (e.g., piezoelectric coefficiency) of most organic‐based materials could not precede that of the inorganic crystals, increasing efforts have been made to synthesize new organic‐based FEMs with competing performance.^[^ ^29^ ^]^ For instance, the effective piezoelectric coefficient of self‐assembled peptide nanotubes in a recent report yields values in excess of 30 pmV^−1^, which performed comparably to the inorganic LiNbO_3_.^[^ ^30^ ^]^ Considering their intrinsic biodegradability and compatibility to the biological systems, native biomolecule‐based ferroelectric biomaterials holds great potentials for their utilizations in biomedical applications.^[^ ^24^, ^27^ ^]^ Here, we list several well‐studied as well as some recently‐developed FEMs, and provide with their typical material characteristics, which may be useful for the bio‐related applications (**Table** **1**).

**Table 1:** Summary of typical properties of some representative FEMs

		Compounds	Spontaneous polarization [*μ*C cm^−2^] ^a)^	Curie temperature [K] ^a)^	Young's modulus, [GPa] ^a)^	Biocompatibility	Biodegradability	Published reports for biomedical applications	Reference
Inorganic		BaTiO_3_	26	396	230	Low toxic	Nondegradable	Extensive	^[^ ^31^ ^]^
		LiNbO_3_	70	891	135	Low toxic	Nondegradable	Extensive	^[^ ^31^, ^32^ ^]^
		Pb(Zr*_x_*Ti_1−_ *_x_*)O_3_	10–18	373–473	130–150	Potential toxicity of metal	Nondegradable	Extensive	^[^ ^33^, ^34^ ^]^
		BiFeO_3_	95	1100	131–170	Low toxic	Nondegradable	Extensive	^[^ ^35^ ^]^
Organic	Polymer	PVDF	≈8	≈400	≈2	Nontoxic	Nondegradable	Extensive	^[^ ^36^ ^]^
		Nylon	≈8	≈450	≈3	Nontoxic	Nondegradable	Rare	^[^ ^37^ ^]^
	Organic perovskites	*N*‐methyl‐*N*′‐diazabicyclo[2.2.2]octonium—NH_4_I_3_	22	448	N.A. ^c)^	N.A. ^c)^	N.A. ^c)^	None	^[^ ^38^ ^]^
	Organic salt	Diisopropylammonium bromide	23	426	50	N.A. ^c)^	Water‐soluble	None	^[^ ^39^ ^]^
	Biomolecules	Glycine	70	N.A. ^c)^	26‐94	Biocompatible ^b)^	Degradable ^b)^	Rare	^[^ ^40^ ^]^
		Elastin	1	570	N.A. ^c)^	Biocompatible ^b)^	Degradable ^b)^	None	^[^ ^15^, ^26^ ^]^
		Peptide (diphenylalanine peptide self‐assemblies)	4	N.A. ^c)^	≈19	Biocompatible ^b)^	Degradable ^b)^	Considerable	^[^ ^27^, ^41^ ^]^
Hybrids	Metal‐organic framework	[Co(II)Cl_3_(H‐MPPA)]	6.8	N.A. ^c)^	N.A. ^c)^	Potential toxicity of metal	N.A. ^c)^	None	^[^ ^38^, ^42^ ^]^
	Organic‐inorganic perovskite	Me_3_NCH_2_ClMnCl_3_	4.0	406	N.A. ^c)^	Potential toxicity of metal	N.A. ^c)^	None	^[^ ^25b^ ^]^

^a)^ Data based on testing on bulk crystals;

^b)^ Considered as biocompatible or biodegradable. However, the corresponding immunological response needs to be evaluated;

^c)^ Data not available.

Up to now, the FEMs available for the intention of biomedical use are still limited (Table 1). One challenge is that most inorganic FEMs are inflexible oxides that undergo brittle deformation, while some of them contain toxic metal ions (e.g., lead and zinc).^[^ ^34^, ^38^ ^]^ In addition, organic‐based FEMs usually possess relatively weak performance, which may further suffer from dysfunction in physiological condition due to their relatively low *T*_c_ or unwanted solubility.^[^ ^25^, ^43^ ^]^ Furthermore, there are difficulties in fabrication techniques for achieving the expecting properties in one material system, for instance, conflicts always exist between piezoelectric coefficiency and flexibility or degradability.

**Figure 2:**
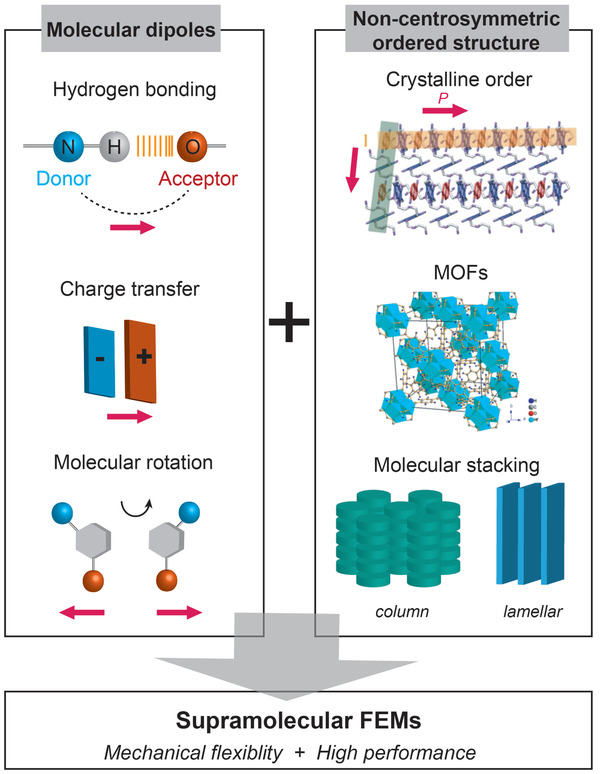
Supramolecular assembly of molecular dipoles to form muti‐functional FEMs. Reproduced with permission.^[^ ^44^ ^]^ Copyright 2019, American Chemical Society. Reproduced with permission.^[^ ^45^ ^]^ Copyright 2019, Wiley‐VCH.

To this end, recent efforts are focused on developing novel strategies and methods for synthesizing FEMs with advanced properties to conquer the above challenges. First, structural requirements identified within inorganic ferroelectrics inspired and enabled the development of organic systems. As shown in **Figure** **2**, molecular dipoles are placed to reside within a noncentrosymmetric ordered structure to form supramolecular FEMs. The bistable dipoles include the utilizations of the oppositely charged electron donor‐acceptor in the charge transfer complex, proton transfer in hydrogen bond as well as the molecular rotation; while the supramolecular assemblies include the use of organic crystalline lattices, porous metal–organic frameworks, and soft materials such as columnar liquid crystals.^[^ ^25^, ^42^, ^43^ ^]^ More information on this topic can be found in recent comprehensive reviews.^[^ ^24^, ^25^ ^]^ In addition, traditional synthetic methods (e.g., solvothermal/hydrothermal, templating, molten salt, and sol–gel methods) for inorganic FEMs in form of nanostructures, thin film or bulk crystals, can be found elsewhere.^[^ ^46^ ^]^

**Figure 3:**
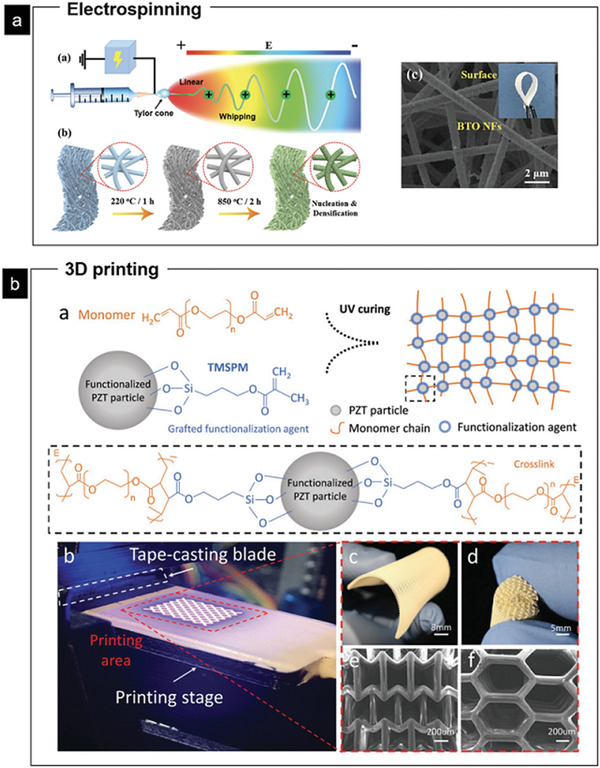
Fabrication of inorganic FEMs using polymer‐processing techniques including electrospinning and 3D printing. a) Schematic of the fabrication processes using sol–gel electrospinning followed by calcination to obtain flexible BTO nanofiber films. Reproduced with permission.^[^ ^48^ ^]^ Copyright 2019, Wiley‐VCH. b) 3D printing of PZT with complex 3D microarchitectures. The functionalized PZT particles are dispersed in the UV‐sensitive monomer matrix. Additive manufactured piezoelectric complex structures with excellent flexibility and fine surface finish are processed via the custom fabrication system. Reproduced with permission.^[^ ^53b^ ^]^ Copyright 2019, Wiley‐VCH.

On the other hand, techniques including solvent‐casting, spin coating, printing technologies, nonsolvent or temperature‐induced phase separation, template removal, freeze drying, and electrospinning, have been successfully used to produce PVDF‐based FEMs with desired morphologies/structures including dense films, porous films, 3D scaffolds, patterned structures, fibers, and spheres (detailed demonstrations of processing techniques for different structures should be found in ref. ^[^ ^47^ ^]^). These reliable methods for processing organic materials can be adapted to develop inorganic FEMs as well. For instance, a scalable strategy has just been reported for the fabrication of 1D ultra‐flexible crystalline BaTiO_3_ nanofibers by a sol–gel electrospinning method (**Figure** **3**a).^[^ ^48^ ^]^ The ceramic fibrous films have a polymer‐like softness of 50 mN, a Young's modulus of 61 MPa, and an elastic strain of 0.9%, meanwhile exhibiting a rapid piezoelectric response time of 80 ms, and an open‐circuit voltage of 1.05 V under a mechanical pressure of 100 kPa.

Additionally, recent effect for making super‐elastic 2D ferroelectric single‐crystal BTO membrane has been achieved via a damage‐free lifting‐off process.^[^ ^49^ ^]^ The freestanding BTO membrane demonstrated a super‐elasticity and ultra‐flexibility due to the dynamic evolution of ferroelectric nanodomains, so that it can undergo an ≈180° folding during an in situ bending test and could be easily integrated with a polymer substrate. Considering the fact that the popular form of FEMs in current application is thin film‐based structure,^[^ ^50^ ^]^ FEMs with 1D^[^ ^46b^ ^]^ or 2D^[^ ^51^ ^]^ morphologies may enable many applications such as flexible sensors, biological generator, and electronic skins (e‐skins).

Furthermore, FEMs with 3D morphologies have been synthesized with the flourishing development of additive manufacturing technology.^[^ ^25^, ^38^, ^52^ ^]^ For instance, advanced 3D printing techniques can make ferroelectric PZT with designed anisotropy and directional piezoelectric response.^[^ ^53^ ^]^ As shown in Figure 3b, the highly concentrated PZT nanoparticulate inclusions were embedded in a light‐sensitive monomer matrix to print 3D porous structures. The polymer modification significantly enhances the piezoelectric coefficients as well as achieves a high flexibility. The printed composites possess high electromechanical sensitivity and structural functionality, as highly sensitive wearables that detect low pressure for wearable devices. Moreover, the organic/inorganic composite FEMs have demonstrated superior performances over each single composition.^[^ ^46^, ^54^ ^]^ Therefore, additive manufacturing of FEMs and FEMs‐based devices with excellent performance has attracted unprecedented attention and will be applied in a wider range of applications in the future.

It is known that the structural characteristics of the extracellular microenvironment are one of the key factors for achieving specific cell responses in vivo. Therefore, by selection and optimization of processing techniques, FEMs can regulate cell behavior through the possibility of configuring specific extracellular morphology as well as providing physical stimuli. For example, Kim et al. studied the response of neural cells to the PVDF‐based electrospun fibers with topological gradient structures.^[^ ^55^ ^]^ These tunable surface topographic constructs, from micropatterns to fiber bundle structures, were obtained by using different shape modified collectors. It is found that the topographical features together with the piezoelectrical cues can not only affect cell growth, but also activate neuron specific cytoskeletal related signaling pathway. The above advancing fabrication techniques offer great opportunities for developing novel FEMs with desired structures and functionalities, as such FEMs featured with diverse morphologies may hold great promise for the field of biomedicine.

The biocompatibility and biodegradability of FEMs are crucial parameters for their medical use as implantable healthcare devices or as tissue engineering scaffolds, where the biocompatible implanted FEMs ideally need to be broken down in the body and eventually removed and replaced by the surrounding tissue after they have served their function. However, as shown in Table 1, most FEMs are nondegradable due to their poor aqueous solubility and pristine inertness to the biological environment. Even more, the chemical composition of some FEMs includes transition metal elements like toxic lead, which may raise long‐term health concerns. Therefore, biomolecular FEMs (e.g., triphenylalanine (FFF) peptide nanoassemblies and glycine crystal),^[^ ^56^ ^]^ which can be digested by enzymes or gradually dissolved in the physiological condition, possess unique potential use as biodegradable healthcare devices, clinical contrast agents, or regenerative scaffolds. For example, Hosseini et al. reported a biocompatible and flexible piezoelectric pressure sensor made of biodegradable glycine and chitosan film.^[^ ^57^ ^]^ The chitosan not only enhanced the flexibility of the brittle glycine crystals, but also controlled the polymorph selectivity of the glycine molecules. Though the composite film could be dissolved in a phosphate‐buffered saline solution within a few days, it is expected that the degradation rates of the biodegradable device can be tunable by encapsulation with a more water‐resistant polymer layer. However, studies have shown that aggregation of biomolecules may invoke potential immune response, therefore, immunological investigation of biomolecular FEMs need to be done before further applications.^[^ ^58^ ^]^

In all, FEMs have broad compositions ranging from inorganic crystals to molecular assemblies. In particular, the development of biomolecular FEMs might bring exciting opportunities to biomedical applications as they have the congenital advantages of interacting actively with the bioelectrical processes in human body. Furthermore, with increasing novel fabrication technologies being developed, the structural morphologies of FEMs could range from nanoscale to bulk size, or from 1D to 3D. Therefore, it is expected that FEMs, with variable compositions and tunable morphologies, have great potential to meet complex practical requirements and find broader applications in the field of biomedicine.

## 4 FEM‐Based Biomedical Applications

Physical stimuli, including electrical, mechanical, and photonic signals, have profound effects on numerous biological processes, therefore FEMs have shown to hold tremendous potentials for a wide variety of biomedical applications in the past few decades and this field is constantly growing. In this section, we attempt to demonstrate how biological substances, that is, biomolecules, cells, and tissues, interact and respond to those FEMs‐mediated physical stimuli, by summarizing recent studies related to biomedical applications.

### 4.1 Biological Response to FEMs‐Mediated Electrical Stimuli

#### 4.1.1 Biological Response to Static Polar Charged Surfaces

**Figure 4:**
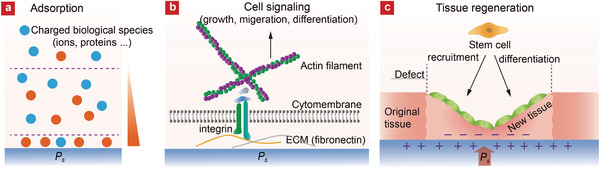
Schematic illustrations of FEMs‐mediated control of a) molecule adsorption, b) cellular behavior, and c) tissue regeneration process by the charged surfaces induced by the spontaneous polarization.

When the charged surfaces induced by the *P*_s_ of FEMs are immersed in biological media (e.g., human plasma), biological species (e.g., ions and protein) are adsorbed on the interface via electrostatic interaction. Near the charged surface this electrostatic interaction force could attract opposite charged species from the medium. The attraction of charged species from the medium to the ferroelectric polar surface makes a layer with an increasing ion/biomolecule concentration due to ions/biomolecules trapped near the surface. A simplified illustration of this phenomenon is shown in **Figure** **4**a. For instance, it has been reported that negatively charged surfaces were able to attract more calcium ions in the simulated body fluid, which could facilitate the accelerated biomineralization in vitro.^[^ ^59^ ^]^ For biomolecules such as protein, they are electrically charged in biological fluid due to adsorbed ions or constituent ionizable surface chemical groups such as hydroxyl carboxylic or amino groups. Therefore, the protein adsorption at charged interfaces is much more complex than simple ions due to the asymmetrical distribution of groups of different charge on the protein surface,^[^ ^60^ ^]^ nevertheless, different protein adsorptions on polar surfaces have been studied. Tarafdar et al. reported higher amount of bovine serum albumin was adsorbed on the positively charged surfaces compared with the negative ones. In a more recent report, the formation of surface calcium phosphates and protein adsorption are considerably enhanced for stainless steel functionalized with a ferroelectric LiTaO_3_ (LT) coatings.^[^ ^61^ ^]^

The preferred (or enhanced) biomolecules adsorption on polar charged surfaces could further activate some important signaling pathways associated with cellular proliferation, differentiation, and function (Figure 4b). Fibronectin, for instance, is an ECM protein that possesses surface bound fragments that could offer binding sites to cells. Weng's group designed P(VDF‐TrFE)‐based ferroelectric films with a wide range of surface potential. They found that the charged surfaces could effectively govern the binding state of the adsorbed fibronectin with integrin and proposed that a full binding state of integrin *α*5*β*1 with fibronectin at an appropriate surface potential induces effective activation of integrin‐mediated FAK/ERK signaling pathway to upregulate cellular osteogenic differentiation of the MC3T3‐E1 cells.^[^ ^62^ ^]^ They further did a similar comprehensive study on the mesenchymal stem cells (MSCs).^[^ ^63^ ^]^ In addition, our previous studies on stem cells cultured on polarized LiNbO_3_ crystal substrates have shown enhanced osteogenic differentiation, as well, though different signaling pathways were proposed.^[^ ^32d^ ^]^ Blazquez‐Castro et al. made an excellent summary of current research studies on cellular response to FEMs along with the mechanisms involved when cells interact with polar surfaces.^[^ ^64^ ^]^

The spontaneous polarization could also provide a build‐in electric field and electric potential near the surface that could be beneficial to the repairing of tissue defects by restoring physiological electrical microenvironment. Deng's group reported the use of BaTiO_3_/P(VDF‐TrFE) composite film to provide a surface potential up to −76.8 mV in the bone defect sites, which conforms to the level of endogenous biopotential. The membranes encouraged the osteogenic differentiation of bone marrow MSCs and the membranes sustainably maintained the electric microenvironment, giving rise to rapid bone regeneration and complete mature bone‐structure formation (Figure 4c).^[^ ^65^ ^]^ A more comprehensive study on the dose–response relationship between surface potential and osteogenesis was further presented.^[^ ^66^ ^]^ Promising results are not limited to bone formation; recent studies have shown that the electrical cues mediated by PVDF‐based scaffolds could not only affect cell growth, induce the intracellular signaling pathway,^[^ ^55^ ^]^ but also exhibit significant electrophysiological, morphological, and functional nerve restoration.^[^ ^67^ ^]^

Taken together, FEMs with built‐in spontaneous polarization electric field can provide static charged surfaces or controlled electric potentials near the surface that are able to regulate biological events. However, there remains a challenging issue that the FEMs used in the above studies are not biodegradable, that is to say, once implanted they will remain in human body for a long time until being removed through an extra surgical operation. Therefore, future direction for developing FEMs with biodegradability would be beneficial to achieve better biomedical performances.

#### 4.1.2 Biological Response to FEMs‐Mediated Dynamic Electrical Stimulation

**Figure 5:**
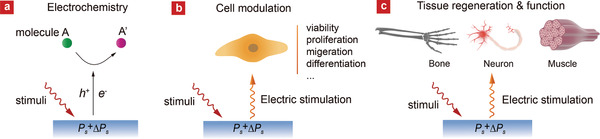
Schematic overview of FEMs‐mediated dynamic electrical stimulation on a) molecules, b) cells, and c) tissues.

Besides the static charged surfaces of FEMs, dynamic electrical signals generated from piezo/pyroelectricity or photovoltaic effects via FEMs can also have profound influences on biological processes (**Figure** **5**). In this dynamic context, both external energy source and internal biological energy can be converted by FEMs to electric energy, which is further involved in biochemical reactions near the interface, bioelectricity‐controlled cell modulation, or tissue function recovery. On the other hand, the electrical responses of FEMs induced by body motion, heartbeat, blood pressure, can serve as excellent indicators of human health conditions, making them next‐generation monitoring e‐devices.

##### Electrochemistry

**Figure 6:**
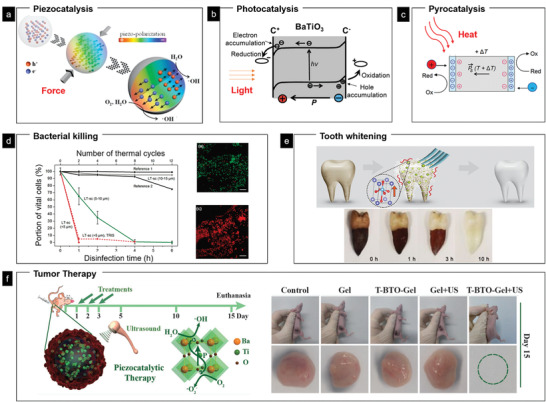
FEMs‐mediated electrochemistry induced by a) mechanical stress,^[^ ^69b^ ^]^ b) photoirradiation (band bending caused by the polarization in BTO and the free photoexcited carriers act jointly to enhance redox chemical reactions),^[^ ^72^ ^]^ or c) thermal excitation.^[^ ^69d^ ^]^ Reproduced with permission.^[^ ^69b^ ^]^ Copyright 2017, Elsevier. Reproduced with permission.^[^ ^72^ ^]^ Copyright 2013, American Chemical Society. d) Antibacterial activity of LT powders of different particle sizes against *E. coli*. are compared (red line, <5 µm; green line 5–10 µm; black lines as references). Fluorescence microscopy images of LIVE/DEAD stains of *E. coli* cultures not subjected to thermal treatment (right upper), and after 1 h thermal treatment in the presence of LT (right below). Reproduced with permission.^[^ ^69d^ ^]^Copyright 2012, American Chemical Society. e) Piezocatalysis of BTO nanoparticles under ultrasonic vibration for tooth whitening and digital images of whitened tooth after 10 h of treatment (below).Reproduced under the terms of the CC‐BY 4.0 license.^[^ ^70^ ^]^Copyright 2020, The Authors, published by Springer Nature. f) The piezocatalytic therapy by BTO‐contained gel combined with US (ultrasound) irradiation remarkably suppress tumor growth in vivo. Reproduced with permission.^[^ ^73^ ^]^ Copyright 2020, Wiley‐VCH.

When FEMs are excited by certain energetic stimulus, electrochemical redox reactions are expected to occur at the material surface due to the generation of separated electron–hole pairs and the collision of them with substance molecules in the physiological medium (Figure 5a). This physical stimuli‐driven electrochemistry has been shown in many reports mainly in the field of catalysis.^[^ ^68^ ^]^ For instance, FEMs, especially FEMs in form of nanocrystals with high surface areas, have been used for the electrochemical reactions (e.g., degradation of organic substances) via perturbations by external fields, including mechanical stress (**Figure** **6**a), temperature (Figure 6b), or photoirradiation (Figure 6c).^[^ ^32^, ^68^, ^69^ ^]^ One of the working mechanisms possibly relies on the stimuli‐induced generation of reactive oxygen species (ROS) such as superoxide anion (O_2_•) or hydroxyl radical (•OH). This ROS generation mechanism has been extensively used for degradation of organic substances and recently utilized toward biomedical applications. One study demonstrated the thermally‐induced antimicrobial activity of nanocrystalline LiNbO_3_ and LT, owing to their pyroelectrocatalytic property, against bacterium *Escherichia coli* in aqueous solutions (Figure 6d).^[^ ^69d^ ^]^ In 2020, Wang et al. reported a novel tooth whitening strategy based on piezoelectrocatalytic effect by using BTO nanoparticles as tooth paste.^[^ ^70^ ^]^ The BTO nanoparticles could produce ROS under ultrasonic vibration so that organic stains on teeth were destroyed, leading to whitened teeth after vibration (Figure 6e). The piezoelectrocatalytic generation of ROS can be further used for tumor eradication. As shown in Figure 6f, Shi and his coworkers employed piezoelectric tetragonal BaTiO3 (T‐BTO) nanoparticles, combined them into a thermosensitive hydrogel and injected the gel (T‐BTO‐Gel) into the 4T1‐tumor xenografts bear on mice. After ultrasound irradiation, the tumor growth was markedly suppressed after intratumoral injection of T‐BTO‐Gel in the first five days after three treatments. In comparison, the tumors in the control groups kept growing to as large as 800 mm^3^. As ROS plays an essential role in regulating various physiological functions, there are great potential areas to be explored for FEMs‐driven electrochemistry to act as stable and controllable therapeutic methodology for biomedicine in the near future.^[^ ^71^ ^]^

##### Electrical Stimulation of Cells

**Figure 7:**
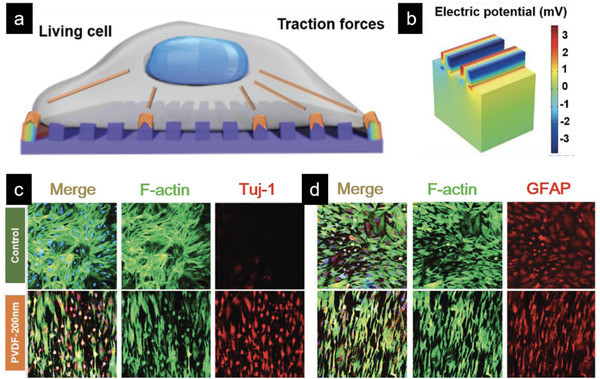
Electrical stimulation of stem cells via cellular movement on PVDF substrate. a) Schematic illustration of inherent cell forces of living cells grown on the surface of PVDF with nanoscale stripe arrays. b) COMSOL simulation of the PVDF of stripe arrays with 200 nm in width generating a maximum positive voltage of 3.4 mV when strained by a tangential force of 10 nN. Immunofluorescent staining of the MSC neuron‐like differentiation on the PVDF substrate after 7 d culture: c) the neuron specific maker Tuj‐1 and d) a neurogliocyte specific maker GFAP. The cell nuclei were stained blue, F‐actin green, Tuj‐1 and GFAP red. Reproduced with permission.^[^ ^36c^ ^]^ Copyright 2019, Wiley‐VCH.

Electricity has long been recognized as an important biophysical factor in living systems, which have inspired numerous investigations to mimic bioelectricity and endogenous electric fields by external electrical stimulation to enhance cellular migration, growth, and differentiation (Figure 6b). Cells could sense and respond to electrical signals through mechanisms including electric filed‐induced change in intercellular calcium flow, membrane protein distribution, alteration of plasma membrane polarization, and so on.^[^ ^74^ ^]^ One unique advantage of FEMs is that the electrical field can be generated without the use of either real electrodes or power suppliers, therefore making them excellent in situ electric generator and stimulator. For example, Li et al. has demonstrated the use of cell migration and traction on PVDF with nanostripe array structures (**Figure** **7**a) to generate a surface piezoelectric potential up to millivolt (Figure 7b), which could locally induce neuron‐like differentiation of the attached MSCs (Figure 7c,d).^[^ ^36c^ ^]^ Promotion of neuronal differentiation of PC12 cells by regulating electrical cue and mechanotransduction in topological gradient PVDF structure was reported in another similar study.^[^ ^75^ ^]^ The FEMs‐enabled self‐stimulation of stem cells by their own movements lead to a novel approach for biomedicine, as it does not require the use of any exogenous growth factors or external stimuli cells normally need for differentiation. This approach may satisfy the need for inductive materials to guide cell commitment, yet its long‐term inductive effect should be taken into consideration where directed cell differentiation is only required in a specific period of time.

External energy can also be utilized to induce electric stimulation via FEMs to cells as well, among which the ultrasound wave is widely used as energy resource due to its deep tissue penetration and ease of tuning in power or frequency. In a pilot study, dynamic stimulation of PC12 cells cultured on PVDF under ultrasound vibration has been shown to activate the cellular calcium channels, thus inducing the neurite differentiation of neurites via a cyclic adenosine monophosphate‐dependent pathway.^[^ ^76^ ^]^ More importantly, the promotive effect of the piezoelectric stimulation of cells on PVDF substrates is comparable to the cells induced by neuronal growth factor, which holds great promise for the development of noninvasive and drug‐free neuroregenerative devices. Moreover, FEMs nanoparticle‐based electric stimulation of neural cells has been reported in terms of induction of calcium and sodium transients, neurite outgrowth, and *β*3‐tubulin expression.^[^ ^77^ ^]^ For example, BTO nanoparticles were exploited as nanotransducers to provide wireless ultrasound‐mediated electrical stimulation of SH‐SY5Y neuron‐like cells, eliciting a significant calcium and sodium fluxes by the activation of voltage‐sensitive channels.^[^ ^78^ ^]^ In a more recent study, Zhao et al. combined BTO nanoparticles with special designed carbon shell, forming the core–shell‐structured nanoparticle, which can be electromagnetized by ultrasound stimulation.^[^ ^79^ ^]^ The electromagnetic fields generated by the composite nanoparticles modulate intracellular calcium signaling to influence synaptic plasticity and control neural behavior, which can serve as a wireless therapeutic candidate for Parkinson's disease. Besides the effect on neurite regulation, the FEMs‐mediated electrical stimulation on promoting osteogenic or myogenic differentiation of stem cells in the field of tissue engineering has drawn extensive attention as well.^[^ ^37^, ^80^ ^]^

In addition, the FEMs‐mediated electrical stimulation can be able to affect cellular viability. It is reported that BTO nanoparticles, which were functionalized to target HER2‐positive breast cancer cells, significantly reduced the cancer cell proliferation via locally delivering an electric stimulation mediated by ultrasounds.^[^ ^81^ ^]^ In other studies related to bacterial cells, the growth or inhibition behavior of certain bacteria species can be tailored through the application of piezoelectric stimuli as well. The positively charged PVDF was found to induce bacterial growth inhibition in planktonic and adhered cells in static conditions, whereas antifouling properties were obtained when mechanical stimuli at 4 Hz was applied.^[^ ^82^ ^]^ It should be noted that electric stimulation may not be the main mechanism to manipulate cell viability, as discussed in Electrochemistry, ROS may affect cell growth as well.

**Figure 8:**
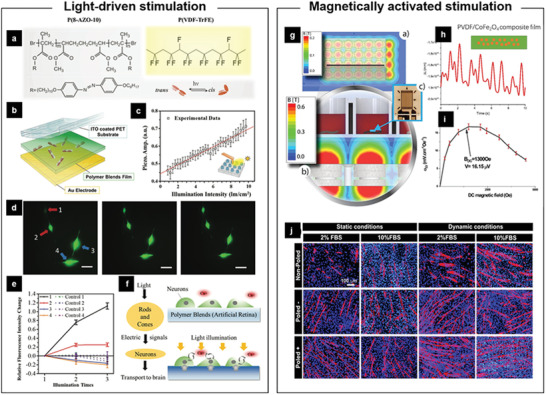
Light‐driven /Magnetically activated piezoelectric stimulation of cells in vitro. a) Molecular structures of azobenzene polymer P(8‐AZO‐10) and its conversion of *trans* and *cis* isomers upon photoirradiation. b) Layout of a ferroelectric polymer arrays as photodetector, consisting of ITO coated PET, 10‐µm‐thick P(VDF‐TrFE)/P(8‐AZO‐10) blends, and Au electrode. c) Profile of piezoresponse amplitude as a function of light intensity. d) Calcium‐ion imaging of PC12 cells cultured on the blend membrane in response to the white LED stimulation. e) The change of fluorescence intensity for the cells seeded on the blend membrane or not. f) Scheme of the signal transduction from the photoreceptor cells (left) or the photodetector to the neuron cells (right). Reproduced with permission.^[^ ^84^ ^]^ Copyright 2016, Wiley‐VCH. g) Illustration of simulated magnetic field and the culture setup for myoblast. h) Measurement mechanical strain induced by the applied dynamic magnetic field with the change of time. i) magnetoelectric response of the CFO/P(VDF‐TrFE) composite film for a DC field from 0 to 5000 Oe at a constant Hac of 1 Oe. j) Immunofluorescent staining of myosin heavy chain after 5 days of C2C12 cells differentiation on the different samples. Reproduced with permission.^[^ ^85^ ^]^ Copyright 2020, American Chemical Society.

In addition to ultrasound, external light‐driven or magnetically controlled electrical stimulation of cells via FEMs has drawn increasing attention. In an early work, necrotic cell death was observed in human tumor cells grown on an iron‐doped lithium niobate substrate after irradiation with visible light, though the underlying mechanism remains unclear.^[^ ^83^ ^]^ In a recent study, photoisomerization of azobenzene dye polymer molecules [P(8‐AZO‐10)] embedded in the P(VDF‐TrFE) membrane has been designed as artificial retina, which triggers electric polarization change upon receiving visible light. The azobenzene‐containing polymer undergoes conversion of *trans* and *cis* isomers by instant photoisomerization, leading to mechanical strain inside the film (**Figure** **8**a,b). Therefore, the photosensitive ferroelectric membrane can convert photo energy to electric signal (Figure 8c) that could be directly transduced to the attached neuron cells leading to induction of calcium flux (Figure 8d–f).^[^ ^84^ ^]^ Considering the importance of cell control via photoregulation and its wide potential applications in the field of biomedicine, the light‐driven electric stimulation mediated by FEMs deserves more attention.

For magnetically controlled electrical stimulation, Ribeiro et al. reported the use of a dynamic magnetic field (Figure 8g) could promote the maturation of myoblasts cultured on a magnetoelectric film in vitro, which was prepared by dispersing magnetostrictive particles (CoFe_2_O_4_, CFO) in a P(VDF‐TrFE) matrix.^[^ ^85^ ^]^ As shown in Figure 8h,i, the CFO/P(VDF‐TrFE) composite film can respond the dynamic magnetic field to generate mechanical strain along with electric signals. Under these dynamic electro‐mechanical stimuli, myoblasts showed enhanced fusion and maturation on both nonpoled and poled (with negative and positive surface charge) samples, while poled samples presented significant higher maturation compared to the nonpoled ones due to the piezoelectric stimulation (Figure 8j). In another study, Liu et al. reported a biohybrid soft micromotor fabricated via the integration of *Streptomyces platensis* with magnetic Fe_3_O_4_ nanoparticles and piezoelectric BTO nanoparticles, which achieved single‐cell targeted motion under a low‐strength rotating magnetic field and then precisely induced the differentiation of the targeted neural stem‐like cell under ultrasound treatment.^[^ ^86^ ^]^ The FEMs and their composite with physical stimuli‐responsive materials have shown great potential as wireless therapeutic candidates, which could be beneficial to the development of tissue engineering and precise nanomedicine.^[^ ^79^, ^87^ ^]^

##### Electrical Stimulation of Tissue/Organs

Promising effects of electrical stimulation on cellular behavior motivate the employment of FEMs for various applications in tissue repair or stimulation for function recovery, particularly in bone defect repair, where electrical stimulation via FEMs has been shown to enhance bone regeneration;^[^ ^88^ ^]^ in skeletal muscle regeneration, promote the myogenic formation and induce muscle extraction;^[^ ^80c^ ^]^ and in neural stimulation, promote neurite sprouting and activate neurite function (Figure 5c).^[^ ^74^, ^77^ ^]^ These approaches have been well‐studied in recent years and there are already some excellent reviews on this topic for readers’ reference.^[^ ^74^, ^77^ ^]^

Recently, harvesting energy directly from the human body offers alternative yet attractive approaches for future medical implants. In particular, motions of the heart and lung provide an inexhaustible source of mechanical energy throughout human lifetime. Though human belong to endotherms, there does exist natural temperature variations in human body caused by exercises, circadian rhythms, menstruation, exposure to cold/hot environments, and illnesses such as fever, can also lead to significant changes in body temperatures.^[^ ^89^ ^]^ Within the airways there are pronounced variations of temperatures from the nares to the lower respiratory tract as well. Therefore, FEMs can provide a feasible route to transduce these human body‐derived mechanical/thermal energies into electric signal. For instance, piezoelectric materials can deform with physiological movements and consequently deliver electrical signals to the charge the implanted batteries or to directly stimulate cells or damaged/dysfunction tissue without the need of an external power source. This sustainable or battery‐less energy supply can be an attractive approach to enable self‐powered stimulation system.

**Figure 9:**
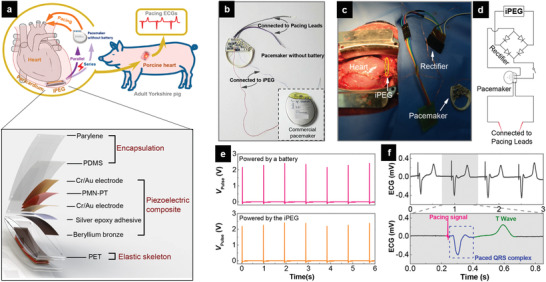
FEM‐powered cardiac pacemaker. a) The schematic illustration for direct powering a real cardiac pacemaker by utilizing energy from a pig's heartbeat (upper) and the symmetry structure of the implantable iPEG (lower). b) Photograph of a commercial cardiac pacemaker with the onboard lithium battery removed. The inset shows the intact pacemaker. c) Photograph of the implanted iPEG in parallel mode, which is connected to the pacemaker through a rectifier. d) Circuit diagram of the in vivo experiment for powering the pacemaker. e) Comparable pulses released by the pacemaker powered by an external battery (upper) or the implanted iPEG (lower). f) Representative pacing electrocardiograms (ECGs) paced by the iPEG‐powered pacemaker. Upper: The uninterrupted pacing ECGs demonstrating the heart are continuously paced. Lower: An enlarged ECG waveform in one intact cardiac cycle demonstrates that the heart is successfully paced by the iPEG‐powered modern full‐function pacemaker. Reproduced with permission.^[^ ^90b^ ^]^ Copyright 2019, American Chemical Society.

Cardiac pacemakers, for instance, use electrical impulses to stimulate the heart muscles and regulate the heartbeat patterns of the patients who suffer sick sinus syndrome or heart block. Surgical procedure is regularly needed (about every 5 years) each time the battery needs to be changed, which are followed with health risks and high costs. In the last decade, FEMs‐based flexible devices have been fabricated to create “micro/nanogenerators,” in order to harvest the available energy from biological activity, including heartbeat or blood flow, into useable electric signals.^[^ ^90^ ^]^ For example, a single‐crystalline Pb(Mg_1/3_Nb_2/3_)O_3_‐PbTiO_3_ (PMN‐PT) film integrated with a flexible polymer substrate was fabricated to form a high‐performance, implantable piezoelectric energy generator (iPEG).^[^ ^90b^ ^]^The device was demonstrated to achieve powering a commercial cardiac pacemaker and restoring the damaged heart function after being implanted into the pericardial sac of an adult Yorkshire swine (**Figure** **9**). In a similar study, the electrical stimuli from PMN‐PT‐based device was directly applied onto the heart muscle even without the use of a real pacemaker.^[^ ^90c^ ^]^ This self‐driven power management demonstrate huge potential for battery‐less medical device, however the parameters of the electric signal (e.g., intensity, frequency, and duration) should be optimized or customized before applying to the patient's tissue or organ, because the biological movements are variable for each individual and different person may have distinct response.

**Figure 10:**
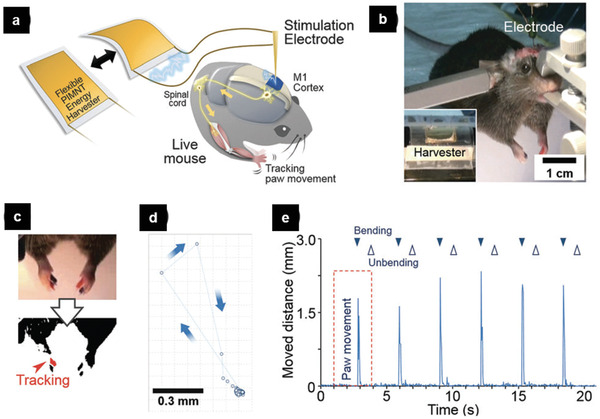
The real‐time self‐powered DBS device. a) An illustration for brain stimulation of mouse using the flexible PIMNT harvester. (M1 Cortex: Primary Motor Cortex) b) The flexible energy harvester is connected to a bipolar stimulation electrode that is localized in the M1 cortex. c) The video‐captured image was adjusted to a binary image, and each marker in the digit was tracked. d) Scattered plot shows the tracked data of the right paw. Each dot is the point of the right paw in each video frame corresponding to the red‐dotted box in (e). e) The distance of paw movement during 6 iterations of electric brain stimulation. Reproduced with permission.^[^ ^91^ ^]^ Copyright 2015, RSC Publishing.

In another example, deep brain stimulation (DBS), is a neurosurgical procedure to stimulate a specific brain area with electric pulses for alleviating various symptoms of neurologic and psychiatric disorders, including epilepsy, Parkinson's disease, essential tremor, and major depression. For the patients who have been injured in the central nervous system, DBS can be employed in terms of low‐energy electrical pulses to artificially achieve the contraction of target muscles and partially restore control over the abnormal body movements. Lee and his co‐workers reported a real‐time self‐powered DBS device to activate specific neurons in brain in a live mouse.^[^ ^91^ ^]^ The device was constructed with Pb(In_1/2_Nb_1/2_)O_3_—Pb(Mg_1/3_Nb_2/3_)O_3_—PbTiO_3_ (PIMNT) thin film integrated on a plastic substrate. It can generate an open‐circuit voltage/a short‐circuit current of 11 V/285 µA, sufficient to meet the high standard of brain stimulation. They successfully stimulated the primary motor (M1) cortex to control body movements, and verified that the functional activation of the M1 cortex induced muscle contraction of the forelimb (**Figure** **10**). This study successfully demonstrates the potential of self‐powered DBS procedure, however future direction of FEMs‐based DBS needs to further integrate the device inside the living system and verify its effectiveness in vivo without the use of the solid electrode needle.

**Figure 11:**
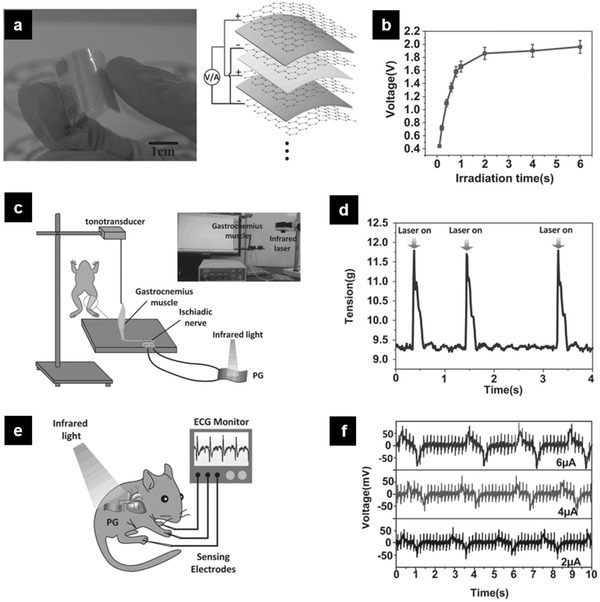
NIR‐driven remote‐manipulative electrical stimulation by the developed pyroelectric generator (PG) device. a) Photograph and scheme of flexible laminated PG device. b) The irradiation time dependence of the open‐circuit voltage. c) Stimulation of a sciatic nerve of a frog. Experimental set‐up for real‐time functional electrical stimulation. d) Tension profile during stimulation by controllable irradiating the device. e) Scheme of the stimulation by the electrical pulse generated by laminated PG device implanted subcutaneously in rat. f) Recorded real‐time ECG in a live rat during heart stimulation by electrical pulse generated by the PG device. The remarkable artificial peaks on the ECG arise from the stimuli clearly showing the validation of the remote‐manipulative stimulation. Reproduced with permission.^[^ ^92^ ^]^ Copyright 2015, Wiley‐VCH.

Though harvesting energy directly from natural processes of the human body provide promising opportunities for powering implantable ferroelectric devices, those biological stimuli are often hard to control in their magnitude, frequency, and timeliness. Therefore, there is great need in the on‐demand controllability of the stimuli as well as the induced electrical signal. Liu et al. proposed a flexible battery‐less implantable pyroelectric generator (PG) device that is constructed by laminated graphene–PVDF–graphene sandwiches.^[^ ^92^ ^]^ The device can generate electrical pulses with controllable amplitude and width under near‐infrared (NIR) irradiation via pyroelectric effect, which can wirelessly stimulate the sciatic nerve of a frog and the heart of a live rat by supplying regulatable electrical pulses on demand (**Figure** **11**). It should be noted that pyroelectric PVDF is also piezoelectric, which may also generate unwanted electric signal when subjected to outside mechanical disturbance. In addition, the power input of the NIR light need to be carefully controlled as the photothermal‐induced overheat shock may damage the surrounding tissue.

### 4.2 Biosensing

FEMs can interact with biological substances and respond to physiological activities, making them promising sensors for the detection of biomolecules, measurement of cell activities, and monitoring human health conditions.

**Figure 12:**
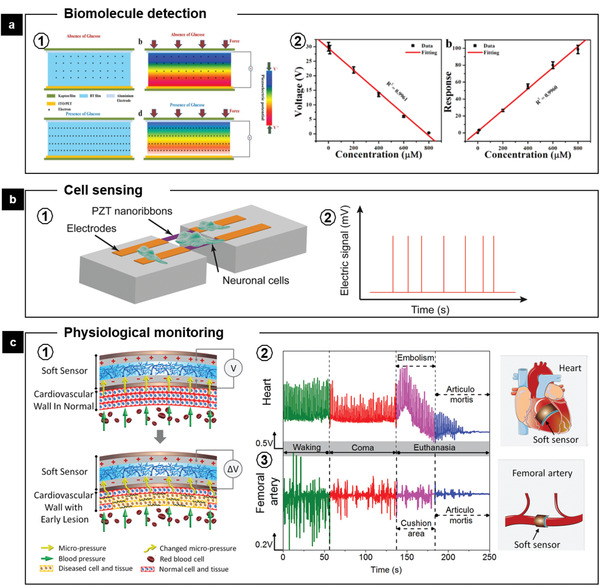
FEMs‐mediated biosensing. a1) Schematic representation of BTO film‐based NG during active biosensing of glucose. a2) Relationship between the piezoelectric output of NG and the concentration of glucose and dependence of the response on the concentration of glucose. Reproduced with permission.^[^ ^93^ ^]^ Copyright 2017, Elsevier. b1) Schematic of the piezoelectric PZT nanoribbon device with cultured neuronal cells. b2) Illustration of the response of piezoelectric nanoribbons to cellular deformations evoked by an applied membrane voltage.^[^ ^94^ ^]^ c1) Schematic of the working principle of the sensor for sensing micropressure changes caused by the early lesion of the cardiovascular wall, which usually led to the changing on the mechanical characteristics or physiological structure of the cardiovascular wall. Output piezoelectric signals of the sensors implanted on c2) heart or c3) femoral artery induced by the cardiovascular elasticity changes of pig at different physiological states. Adapted with permission.^[^ ^98^ ^]^ Copyright (2019) American Chemical Society.

For the detection of biomolecules, Sophia et al. developed a BTO nanoparticle film‐based device for active sensing of glucose molecules (**Figure** **12**a).^[^ ^93^ ^]^ The glucose molecules adsorbed on the ferroelectric surface could act as a gate potential, and the field effect eventually varies the screening effect of free‐carriers on the piezoelectric output. This piezoelectric biosensing achieved detecting concentrations of glucose up to 800 *μ* m with a limit of detection at 10 *μ* m (Figure 12a), which is not yet comparable to the existed detection techniques. Therefore, there is still a long way to go and plentiful room to explore in order to develop FEMs‐based detection at molecular level.

Examining mechanical response of cells to electric stimulation can provide us basic understand of cellular physiology and biology. Nguyen et al. successfully used PZT nanoribbons to maximize the electromechanical effect for measuring mechanical deformations on a nanometer scale (Figure 12b).^[^ ^94^ ^]^ Distinguishable output signals from suspended PZT nanoribbons were recorded simultaneously with the deformation of PC12 cells stimulated by applied membrane voltage. A membrane voltage change of 120 mV induced a cellular force of 1.6 nN on the freestanding PZT thin films when a cellular deformation of 0.5 nm influenced a single PZT ribbon. The PZT nanoribbons provide a novel stage for measuring and recording small signals from cells. In addition, some pilot studies have also been reported to utilize the pyroelectric effect for sensing molecule and cell activities as well.^[^ ^95^ ^]^ Considering the fact that the magnitude of energy at either molecular or cellular level is quite small, their corresponding interactions with FEMs are even more difficult to be detected as well. Though the performance of these proof‐of‐concept sensors need to be further improved, there is still great potential for developing novel FEMs‐based sensors targeted at tissue or organ level.

Over the last decades, biosensors targeted to specific tissues or organs have demonstrated promising capabilities for physiological monitoring, disease diagnosis, and health condition assessment.^[^ ^96^ ^]^ One typical representative application is the emerging e‐skin.^[^ ^97^ ^]^ For instance, Park et al. fabricated a polymer composite film composed of PVDF and reduced graphene oxide with fingerprint‐like patterns and interlocked microstructures, which can enhance the piezoelectric, pyroelectric, and piezoresistive sensing of static and dynamic mechanothermal signals.^[^ ^97a^ ^]^ The e‐skin successfully perceived artery pulse pressure as well as skin temperature simultaneously. FEMs‐based biosensors have shown great potential for diagnostics of diseases as well. Li et al. reported a soft sensor fabricated with PVDF/hydroxylamine hydrochloride (HHE) composite nanofibers (Figure 12c).^[^ ^98^ ^]^ HHE polymer chains were incorporated to the control the spatial uniaxial orientation of PVDF chains to increase the *β*‐phase content. As a result, the PVDF/HEE sensor obtained an ultrahigh detecting sensitivity and accuracy to harvest micropressure changes at the outside of cardiovascular walls. The real‐time output piezoelectric signals, therefore, synchronously distinguish changes of cardiovascular elasticity and achieve early assessment and diagnosis of thrombosis and atherosclerosis. This strategy could be suggestive for diagnose of other diseases as well.

### 4.3 Biological Response to FEMs‐Mediated Mechanical Stimuli

Mechanical stimuli are important biophysical parameters that have long been recognized to able to regulate numerous essential biological processes. The use of external mechanical stimuli (e.g., ultrasound), have already achieved numerous applications in the biomedical field for both diagnostic and therapeutic purposes.^[^ ^99^ ^]^ FEMs, especially PZT and LN crystal, possess excellent ability to transduce electrical pulses input to well‐controlled mechanical wave at the desired frequency (Hz–MHz) based on the inverse‐piezoelectric effect, making them remarkable mechanical stimuli generating platform for cell manipulation and physiological intervention. Rather than explaining the well‐established applications like ultrasound imaging, in this section, we intend to showcase some recent undergoing studies on how FEMs‐generated mechanical stimuli interact with and manipulate biological substances, and to discuss their potential use in biomedicine.

#### 4.3.1 Mechanical Manipulation of Bioparticles

**Figure 13:**
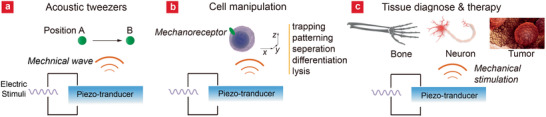
Schematic overview of FEMs‐mediated mechanical manipulation or stimulation on a) molecules, b) cells, and c) tissues.

Mechanical wave is able to control the motions of biomolecules or bioparticles in aqueous environment. The so‐called “acoustic tweezers” (**Figure** **13**a) employ acoustic waves to manipulate bioparticles with size in diameter ranging from nanometer (extracellular vesicles) to millimeter (multicellular organisms), and to move across over five orders of magnitude in length (10^−7^ to 10^−2^ m).^[^ ^99b^ ^]^ For example, the manipulation of acoustic tweezers at single‐molecule level have enabled the development of acoustic force spectroscopy, which uses piezo‐generated acoustic forces to stretch multiple molecules individually tethered between a surface and a microsphere.^[^ ^100^ ^]^ By comparing the displacement of the bead with the magnitude of the applied force, insights into protein‐DNA and protein–protein interactions can be obtained by tracking the force‐extension profile.

**Figure 14:**
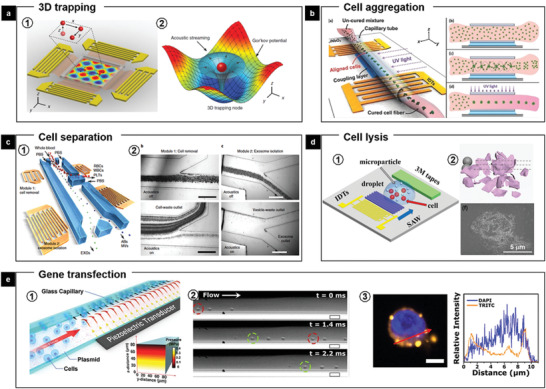
FEMs‐mediated manipulation of bioparticles including cells. a) Two pairs of IDTs are integrated with LiNbO_3_ crystal substrate to generate a planar standing‐wave field (a1). Numerical simulation results show the mapping of the acoustic field around a single particle (a2). Reproduced with permission.^[^ ^101^ ^]^ Copyright 2016, National Academy of Sciences b) Schematic representation of generating a patterned cell fiber in the perpendicular orientation using SAWs. Reproduced with permission.^[^ ^102^ ^]^ Copyright 2016, Wiley‐VCH. c) Acoustic isolation of exosomes from whole blood. Images were taken under a microscope at different separation modules. Scale bars, 500 µm. Reproduced with permission.^[^ ^103^ ^]^ National Academy of Sciences. d1) A mechanical cell lysis device based on a SAW microchip. Schematic and d2) SEM image of the cell–magnetic microparticle collision model and a broken cell. Reproduced with permission.^[^ ^32b^ ^]^ Copyright 2019. Wiley‐VCH. e1) Schematic of the device components, where target cells undergo acoustofluidic treatment via flow through a glass capillary over a piezoelectric transducer. e2) Cells are observed to localize against a capillary wall and are pushed forward by laminar flow. e3) Intracellular delivery with fluorescently labeled DNA (TRITC channels show fluorescence signal of Cy3‐labeled DNA at the cell membrane, cytosol, and nucleus for acoustic‐treated cells). Reproduced with permission.^[^ ^104^ ^]^ Copyright 2016, National Academy of Sciences.

Over the past years, the capabilities of acoustic tweezers have expanded from simplistic particle trapping to precise manipulation of cells and organisms in three dimensions. In 2016, Huang's group presented the use of surface acoustic waves (SAW) to create 3D trapping nodes for the manipulation of microparticles and cells along three mutually orthogonal axes.^[^ ^101^ ^]^ A 2D displacement field on a LiNbO_3_ substrate was created by superimposing two mutually orthogonal pairs of interdigital transducers (IDTs). Each pair of IDTs was individually connected to a double‐channel radio‐frequency signal generator and two amplifiers. The acoustic waves propagated in the fluid, reflected by the chamber walls, and established a 3D, differential Gor'kov potential field, producing 3D trapping nodes within the chamber (**Figure** **14**a). 3D printing of living cells onto a substrate with customized cell patterns was further performed in this work. Moreover, 3D trapping of cells within a photosensitive hydrogel fiber was achieved to mimic physiological cell arrangement in tissues. As shown in Figure 14b, cells aggregates were formed into patterns in the hydrogel within a tube and the aligned cell–polymer matrix was polymerized under UV light to form patterned cell fibers.^[^ ^102^ ^]^ These cell fibers could further be manipulated into complex architectures, demonstrating feasibility for organ printing or tissue engineering applications.

Furthermore, recent advances have led to making reconfigured acoustic tweezers that are capable of separating bioparticles in complex solutions. Huang's group again combined acoustic tweezers with microfluidics on the LiNbO_3_ substrate so that on‐chip technology, it is capable of isolating exosomes or other types of extracellular vesicles, directly from undiluted whole‐blood samples in an automated fashion (Figure 14c).^[^ ^103^ ^]^ In another study, clinical samples of circulating tumor cells from breast cancer patients were successfully separated via an acoustic‐based microfluidic device in a high‐throughput manner.^[^ ^92^ ^]^ The acoustic‐based cell separation is the only active separation technique that can differentiate cells based on their distinct physical characteristics, and possesses many advantages including minimal damage to cells, no need for further cell modification or labeling and no special requirement for cell culture medium.

The particle movement in cell suspension induced by mechanical wave could also lead to collisions between cells and particles, which can be used for cell lysis. In 2019, our group introduced a new mechanical cell lysis method based on a SAW microchip (Figure 14d).^[^ ^32b^ ^]^ It consists of a piece of LiNbO_3_ crystal substrate, IDTs and 3M Magic Tapes. When a biofluid droplet containing cells and microparticles is dropped on the surface of the working microchip, the cells and microparticles are accelerated by the acoustic stream and collide with each other, which disrupt the cell membrane structure and function. This technique provides an on‐chip cell treatment platform for potential analysis of important biomarkers contained in mall‐volume cell droplet.

In another example, acoustic waves were used for pore formation and permeabilization of cell membranes, based on which Weiss et al. demonstrated the acoustofluidic sonoporation device containing PZT substrate as piezoelectric transducer to deliver plasmids to human hematopoietic stem and progenitor cells (Figure 14e).^[^ ^104^ ^]^ The cells under acoustofluidic conditions experience a combination of the shearing force induced by microscale acoustic streaming and the acoustic radiation force that pushes the cells to the microcapillary wall, where membrane leaflets cyclically expand and contract, which results in increased cellular deformation, pore formation, and thus membrane permeability. This acoustofluidic‐mediated approach achieved fast and efficient intracellular delivery of an enhanced green fluorescent protein‐expressing plasmid to cells at a scalable throughput of 200 000 cells per min in a single channel, holding huge promise for cell engineering in a large‐scale manner needed in real applications.

#### 4.3.2 Mechanical Stimulation of Cells

Cells have the ability to sense their local microenvironment and communicate through mechanical cues to regulate cell fate and cell behavior.^[^ ^105^ ^]^ Mechanical stimulation could lead to alterations in cell morphology, changes in cell signaling, and gene transcription via activation of certain mechanoreceptors such as piezochannels (Figure 13b).^[^ ^106^ ^]^ Mechanotransduction is the ability of cells to convert mechanical forces in their environment to biochemical signaling. Studies have shown external mechanical stimulus generated from FEMs to alter cellular responses in both endothelial and MSCs, particularly in increased proliferation rate and induced osteogenesis, respectively.^[^ ^107^ ^]^ Dalby et al. reported the use of nanoscale sinusoidal mechanotransductive protocols (10–14 nm displacements at 1 kHz frequency via piezo actuator), to promote osteoblastogenesis in human mesenchymal stem cell cultures.^[^ ^108^ ^]^ The “nanokicking” platform provides a novel culture substrate system to control stem cell fate in vitro without using soluble factors or complex media formulations. This actuator‐based technique requires direct contact between cells and the vibrating interface, therefore how to transfer this platform to in vivo practice remains challenging.

#### 4.3.3 Mechanical Stimulation of Tissue/Organs

**Figure 15:**
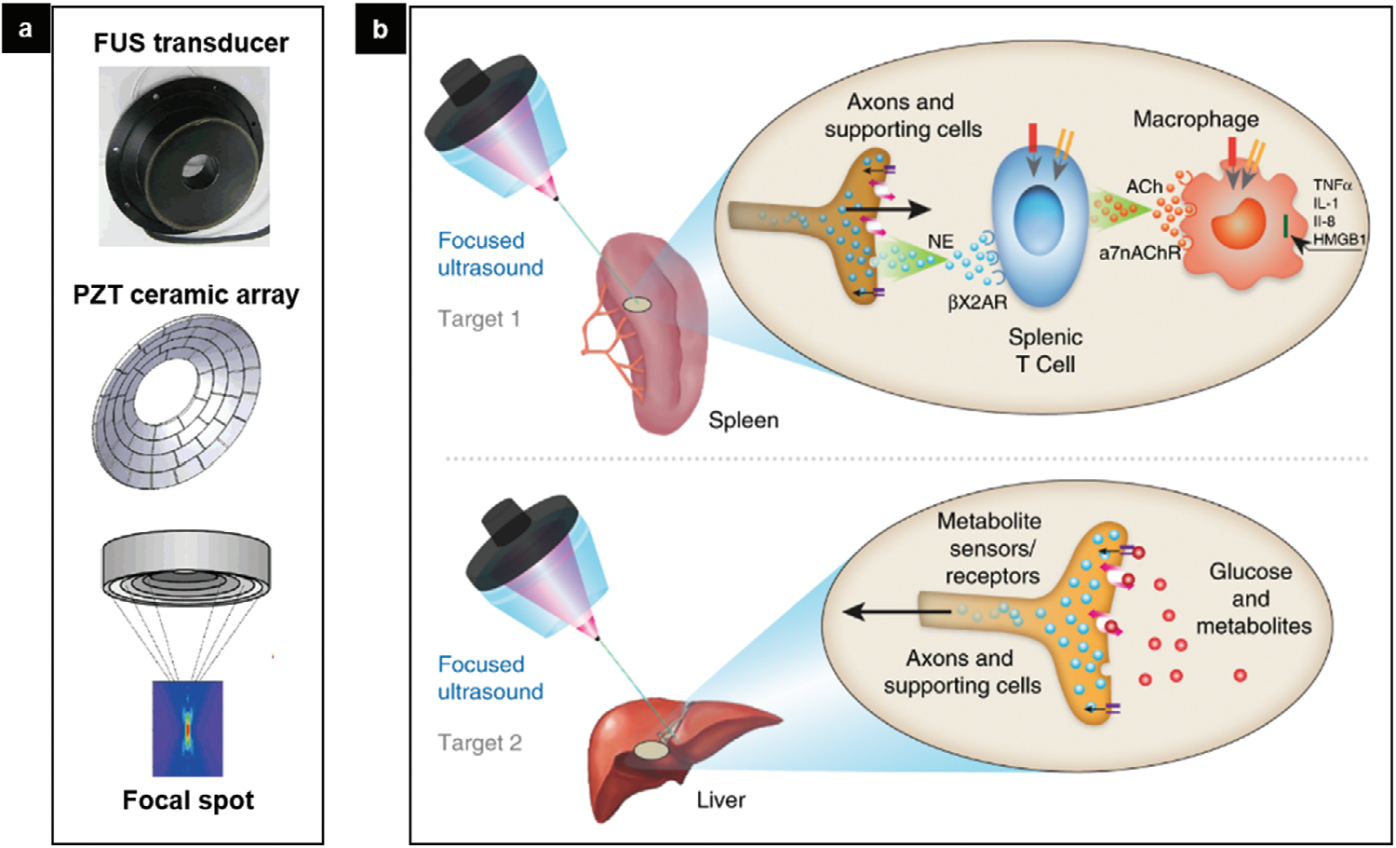
FUS‐enabled neuromodulation. a) FUS is constructed from concaved PZT ceramic array. b) A schematic of precision organ‐based neuromodulation in which the innervation points of known axonal populations are targeted for stimulation using pulsed FUS. Targets investigated herein include innervation points within the spleen and sensory terminals within the liver. FUS stimulation is shown to reduce cytokine response to endotoxin via activating the cholinergic anti‐inflammatory pathway (upper) and the hepatic FUS stimulation is shown to modulate pathways that regulate blood glucose (below). Reproduced with permission.^[^ ^109^ ^]^ Copyright 2019, Springer Nature.

Ultrasound therapy has been widely used as alternative noncontact treatments for tissue injuries like muscle strains or bone fractures (Figure 13c). Those piezoelectric crystals (e.g., PZT) hidden inside the ultrasound device are able to generate and deliver soundwaves with controlled intensities and frequencies. Despite ultrasound therapy suffers from variable therapeutic outcome, recent efforts on precise intervention using focused ultrasound (FUS) demonstrated a promising way to noninvasively deliver mechanical forces into deep tissues. As the focusing of the acoustic waves is achieved through constructive interference of the incident waves via concaved PZT ceramic array (**Figure** **15**a), a focal spot can be formed at depth within the tissue without affecting cells along the propagation path closer to the transducer. One representative application of FUS is the rapidly growing field of ultrasonic neuromodulation, in which low‐intensity ultrasound is delivered to nervous system tissue, resulting in transient modulation of neural activity. Recently, FUS is first applied to spleen to modulate the cholinergic anti‐inflammatory pathway (Figure 15b).^[^ ^109^ ^]^ The study showed reduction in cytokine response to endotoxin to the same levels as implant‐based vagus nerve stimulation. The hepatic ultrasound stimulation is further shown to modulate pathways that regulate blood glucose when targeting specific sub‐organ locations known to contain glucose sensory neurons. This noninvasive sub‐organ stimulation via FUS generated from configured piezoelectric array demonstrates site‐selective neuromodulation to regulate specific physiological functions, which holds great potential for future noninvasive precise therapy.

### 4.4 FEMs for Bioimaging and Phototherapeutics

The ability of FEMs to emit light under physical stimuli enables them potential tools for bioimaging. One main working mechanism of FEMs‐enabled imaging relies on the nonlinear optics. The nonlinear microscopy based on tunable Ti:Sapphire pulsed laser has been well‐developed for bioimaging in recent decades, with advantages such as deep tissue penetration, superresolution, and reduced photodamage. As such, there are urgent needs for photostable imaging probes with absorption bands in the NIR and emission with narrow band width. Fluorescence‐based labels including quantum dots,^[^ ^110^ ^]^ upconverting nanoparticles,^[^ ^111^ ^]^ and aggregation‐induced emission nanoparticles^[^ ^112^ ^]^ have demonstrated excellent optical properties toward this end. FEMs nanoparticles, together with other inherently nonlinear nanoparticles, go with a completely different nonlinear approach by exterting an optical contrast mechanism via the harmonic generation process. Unlike fluorescent probes with classic absorption‐emission mechanism, they possess unique features such as nonbleaching, nonblinking, nonsaturation at high illumination, tunable excitation/emission wavelength, and narrow emission band. With this unique combination of advantageous properties, FEMs‐based nanoprobes could potentially address the challenges imposed by traditional fluorescent probes. In a pilot study, Pantazis et al. demonstrated that the SHG nanoprobes (e.g., BTO nanocrystals) possessed superior signal‐to‐noise ratio compared with quantum dots, and excellent long‐term photostability as well as targeting specificity when imaged after in vivo injections into a zebrafish model.^[^ ^113^ ^]^ They also studied the wavelength‐dependence of the SHG signal from the BTO, compared with ZnO and SiC nanoparticles, which can be further exploited for multi‐probe imaging. Later, Staedler et al. conducted a survey of some typical harmonic nanoparticles including LiNbO_3_, BaTiO_3_, KTiOPO_4_, and KNbO_3_.^[^ ^31a^ ^]^ They compared their ensemble nonlinear optical properties in terms of SHG efficiency and cytotoxicity on some human cancerous and normal tissue cell lines. It was concluded that most of these FEMs‐based nanoprobes are considered as nontoxic and their individual harmonic conversion efficiency can be readily related to that of the corresponding bulk materials and that the harmonic responses of the different samples are very similar.

**Figure 16:**
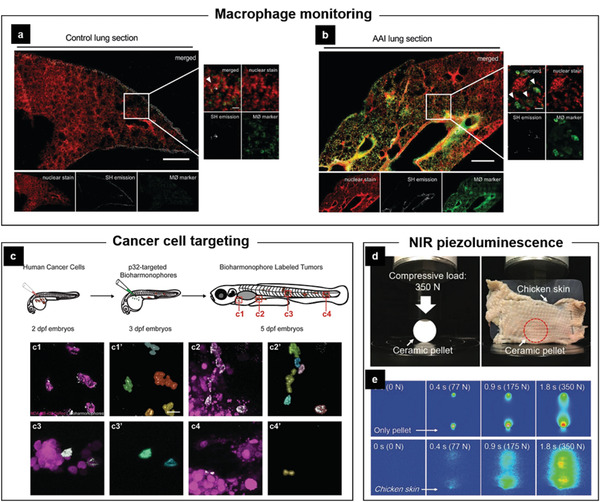
FEMs‐mediated bioimaging for macrophage, cancer cell targeting monitoring, and deep tissue NIR piezoluminescence. a–b) SHG imaging of serum‐covered BFO nanoparticles phagocytosed by native macrophages. Representative two‐photon confocal microscope images of 40 µm lung tissue sections from AAI and control mice stained for the M2 macrophage marker ECF‐L (MΦ). Scale bar represents 500 µm, and in the zoomed pictures 20 µm. Reproduced with permission.^[^ ^35a^ ^]^ Copyright 2018, Wiley‐VCH. c) Schematic showing cancer cell injection of 2 dpf zebrafish embryos followed by injection of p35‐coated FFF peptides nanoparticles into the Duct of Cuvier (DoC) of 3dpf (3 days post fertilization) zebrafish embryos and subsequent fluorescence and SHG imaging at 5 dpf. Individual panels showing the images of labeled cancer cells with the details of bioharmonophore (white) labeling down to single cancer cells (magenta) in solid tumors (c1–4). Colored cell boundary reconstruction of targeted cancer cells using the bioharmonophore SHG signal (c1’–4’). Scale bar, 15 µm. Reproduced with permission.^[^ ^56^ ^]^Copyright 2019, The Authors. d) Sr_3_Sn2O_7_:Nd^3+^ (SSN) ceramic pellet in the material mechanical testing machine (CCD images, left) and blocked with a chicken skin tissue. e) Sequence of images recorded using an InGaAs‐CCD camera for SSN applied during the load cycle of 350 N at a rate of 3 mm min^−1^ showing the real‐time images of NIR light distribution. Reproduced with permission.^[^ ^114^ ^]^ Copyright 2020, Weily‐VCH.

Very recently, Ramos‐Gomes and co‐workers used BFO nanoparticles to monitor macrophages in the lungs of mice suffering from allergic airway inflammation (AAI). Under a two‐photon laser scanning microscope, the bright SHG signals emitted from BFO can detect and track macrophages in thick lung tissues at high resolution, with excellent signal‐to‐noise ratio and minimal background fluorescence.^[^ ^35a^ ^]^ **Figure** **16** shows the immunofluorescence and SHG signals of agarose lung sections explanted 24 h after instillation of BFO nanoparticles, which are located in alveolar and bronchial eosinophil chemotactic factor‐L (ECF‐L)‐positive immune cells in both control and AAI lungs. ECF‐L is a marker used to identify M2 macrophages (MΦ), abundantly expressed in allergic inflammation. Stitched mosaic images of AAI lung sections show significantly higher numbers of ECF‐L+ cells containing BFO around bronchia and blood vessels (Figure 16a) than those of control lungs, where the cells are fewer and more spread around the tissue (Figure 16b). This ex vivo SHG imaging approach provides novel information about the interaction of macrophages with cells and the ECM in lung disease. However, the cell labeling of this work depend on the phagocytosis of macrophages rather than specific recognition between the SHG label and the targeted cells, therefore, future work should expand the targeting of the FEMs‐based nonlinear probes to different cell types.

Until now, there are only a few studies reporting the use of FEMs‐based harmonic nanoparticles for in vivo real‐time imaging since the first work demonstrated by Pantazis and co‐workers back in 2010.^[^ ^113^ ^]^ They directly injected BTO nanoparticles conjugated with Cy5‐tagged secondary antibodies into zygote stage zebrafish embryos, which were then imaged under excitation at 820 nm several days after the injection. Later on, R. Grange et al. demonstrated the detection of BTO nanoparticles through a living mouse tail tissue with depth up to 120 µm.^[^ ^115^ ^]^ Ideally, imaging probes for biomedical applications should be degraded in vivo and the by‐product not lead to any long‐term toxicological consequences. In a latest BioRxiv preprint, Pantazis et al. reported a biodegradable SHG nanoprobes based on FFF peptide nanoassemblies named “bioharmonophores”.^[^ ^56^ ^]^ When functionalized with tumor cell targeting ligands, these bioharmonophores can detect single cancer cells with increased labeling efficiency and high sensitivity in zebrafish embryos in vivo (Figure 16c). Despite the preliminary success in zebrafish or mouse model, it is in urgent need to investigate these nonlinear probes in a larger animal model, though configuration of such imaging platform would be quite challenging.

In addition to nonlinear optics, piezoluminescence is another novel mechanism of FEMs for imaging purpose. Piezoluminescence occurs during elastic deformation of a piezoelectric mechanoluminescence (ML) material, which can exhibit mechano‐electro‐optoelectronic conversion and ML emission under mechanical stimuli. In 2020, Tu et al. successfully developed a low‐stress triggered NIR piezoluminescent material (Sr_3_Sn_2_O_7_ doped with rare earth Nd^3+^ ions, SSN) that exhibit a high ML sensitivity.^[^ ^114^ ^]^ Strong intensity of the piezoluminescence of SSN was observed over a broad range of NIR wavelengths (800–1500 nm) due to the 4f–4f transition in Nd^3+^. As shown in Figure 16d,e, the stress induced NIR emission of the SSN pellet exhibited significant transmission through the chicken skin tissue and the intensity increased dynamically during load application, indicating a dynamic variation of stress concentration. The results suggest that SSN can function as a novel biomechanical probe for deep‐imaging of labeled structures in tissue. Future work on demonstration of this material for detecting dynamical stress distributions of implanted biomaterials in load‐bearing bones or joints within animal models is urgently expected.

**Figure 17:**
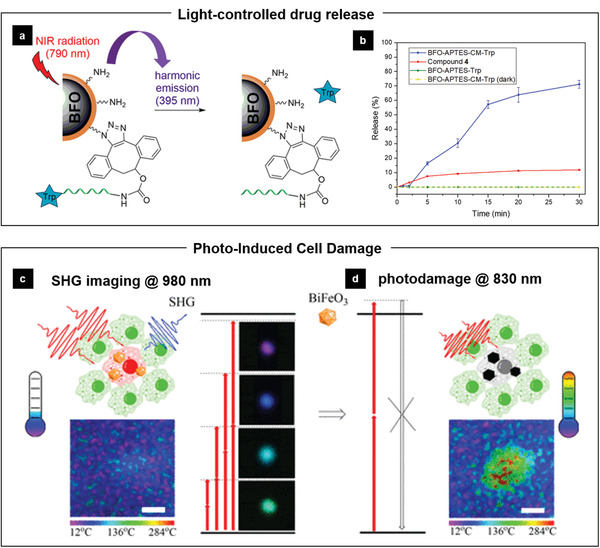
SHG‐mediated drug release and cell damage. a) NIR light‐triggered drug release of Trp from functionalized BFO nanoparticles mediated by SHG process. b) Trp release profiles following irradiation of BFO nanoparticles conjugated with coumarin (CM) as photocleavable linker and Trp as a model molecular cargo (blue curve) with Ti:sapphire pulsed laser system at 790 nm. Reproduced with permission.^[^ ^35c^ ^]^ Copyright 2020, American Chemical Society. c) BFO nanoparticles show SHG emission excited at 980 nm, while d) lead to photodamage to cells at 830 nm. Reproduced with permission.^[^ ^118^ ^]^ Copyright 2020, American Chemical Society.

In addition to bioimaging, a series of application examples from the recent literatures are reported, ranging from in vitro cell labeling and monitoring,^[^ ^116^ ^]^ cell tracking,^[^ ^35a^ ^]^ in vivo imaging,^[^ ^31^, ^117^ ^]^ to the example of light‐controlled release of drug molecules,^[^ ^35c^ ^]^ and photo‐induced cell damage.^[^ ^118^ ^]^ For instance, Vuilleumier et al. presented a BFO‐based nanoplatform for NIR light‐triggered release of molecular cargos.^[^ ^35c^ ^]^ BFO nanoparticles were covalently conjugated to a photo‐responsive tether based on coumarin and L‐tryptophan (Trp) as a model molecular cargo. Upon femtosecond pulsed irradiation at 790 nm, Trp was efficiently released from the particle surface upon NIR irradiation as the SHG emission of BFO at 395 nm induced the photocleavage of the carbamate linkage (**Figure** **17**a,b). Since the wavelength of harmonic emission can be controlled by simply tuning the laser excitation wavelength, further potential applications such as sequential on‐demand release of different molecular cargos might be achieved by conjugating different caging groups sensitive to distinct excitation wavelengths.

Although the harmonic generation process does not directly originate from photon absorption, the harmonic emission can lead to multiphoton excitation or linear re‐absorption by the particles, resulting in a rise in temperature due to the resonant effect. For instance, the band‐gap of BFO nanoparticles is approximately centered at 450 nm, therefore under laser irradiation with wavelengths at NIR‐I region (e.g., 830 nm^[^ ^118^ ^]^), the photo‐interaction of BFO upon excitation at high irradiance obtained a temperature increase to over 100 °C due to resonant interaction in this spectral region. When BFO nanoparticles excited at 980 nm, on the other hand, they showed no obvious rise in temperature and worked as SHG reporters (Figure 17c). The resonant interaction induced thermal effect could result in photo‐induced damage of cells labeled by BFO (Figure 17d). This work demonstrates the multiple working modes of harmonic FEMs as biolabels, since they can be used for both diagnostics (imaging) and treatment (cell disruption) by just tuning the laser wavelengths.

Moreover, the harmonic emission from the FEMs can reach deep ultraviolet (DUV) region, where photo radiation could directly interact with bioactive molecules such as nuclear DNA, whose absorption bands peak around 260 nm. Staedler et al. proposed the use of the pulsed visible light (540 nm) and BFO nanoparticles to generate direct DUV radiation (270 nm) in situ on the particle‐associated human‐derived cancer cells, which could lead to double‐strand breaks in the DNA and cell apoptosis.^[^ ^119^ ^]^ The strategy offers a new way to interact with DNA of malignant cells in absence of photosensitizing molecules and avoid risk of spontaneous activation by natural or artificial light sources other than pulsed femtosecond lasers. However, off‐target safety needs to be taken into consideration in this scenario as the harmonic nanoparticles might at the same time expose the surrounding cells at risk.

## 5 Challenges and Outlook

Recent advances in physical biology and material science have sparked interest in the physical stimuli‐mediated regulation of biological process for controlling physiological behaviors at levels of molecule, cell, and tissue. FEMs, which can sense and convert mechanical, thermal, electrical, and optical energy, possess versatile capabilities to interact with many biological processes, thus demonstrating great potential to work for biomedicine. In this Review, we briefly introduce the working mechanisms of FEMs responding to different physical stimuli, summarize current‐available FEMs along with some recently‐developed FEMs, and then emphasize on highlighting recent advances of FEMs applied in the field of biomedicine.

Recent development of FEMs for biomedical applications discussed in this review is summarized in **Table** **2**. Despite these encouraging achievements, the biomedical application of FEMs still involves several challenges. First, there is a huge gap between the material science of FEMs and their biomedical applications. On one hand, as discussed in Section 3, material scientists have already made great process in designing novel FEMs and developing various fabrication techniques. On the other hand, FEMs used in most of current studies are limited to inorganic crystals like BTO or organic polymers like PVDF and there is urgent need to develop FEMs with customized structures, functionalities or properties. For instance, it would be ideal for FEMs implants to be absorptive after serving their function in human body so that there is no need for unnecessary surgery. But only few organic molecular materials among FEMs are potentially considered as biodegradable. Other properties including flexibility for e‐skin, bioactivity for implants, and high nonlinear co‐efficiency for bioimaging, would also be desired. Therefore, more effects from both material and biology communities need to be investigated to fill this gap. Whereas the beginning of FEMs‐mediated biomedical applications focused on certain crystal or polymer, future work may broaden this field by exploring and taking advantages of materials and systems established elsewhere, as well as by collaborating across disciplines.

**Table 2:** Summary of representative biomedical applications of FEMs

	Applications	Representative FEMs	Working mechanisms	Refs
Molecule level	Biomineralization	PVDF, LiTaO_3_ film	Spontaneous polarization induced surface charge	^[^ ^59^, ^61^ ^]^
	Protein adsorption	PVDF, LiTaO_3_ film	Spontaneous polarization induced surface charge	^[^ ^61^, ^62^ ^]^
	Biomolecule detection	BTO nanoparticle film	Piezoelectric effect	^[^ ^93^ ^]^
	Acoustic force spectroscopy	Piezo‐component	Inverse‐piezoelectric effect	^[^ ^100^ ^]^
	Light‐triggered drug release	BiFeO_3_ nanoparticles	SHG	^[^ ^35c^ ^]^
Cell level	Osteogentic differentiation	P(VDF‐TrFE), LiNbO_3_ substrate	Spontaneous polarization induced surface charge	^[^ ^32^, ^62^, ^63^ ^]^
		Nylon nanoparticles	Piezoelectrical stimulation	^[^ ^37a^ ^]^
		PVDF/Ag‐BTO scaffolds	Piezoelectrical stimulation	^[^ ^80d^ ^]^
		Commercial piezotranducer	Inverse‐piezoelectric mechanical stimulation	^[^ ^107^, ^108^ ^]^
	Neuronal differentiation	PVDF/BTO/multiwall carbon nanotubes fibrous scaffolds	Spontaneous polarization induced surface charge	^[^ ^55^ ^]^
		PVDF with nanostripe array	Piezoelectrical stimulation	^[^ ^36c^ ^]^
		BTO nanoparticles	Piezoelectrical stimulation	^[^ ^78^ ^]^
	Myoblast maturation	CoFe_2_O_4_/P(VDF‐TrFE) composite film	Magnetically activated mechanical/piezoelectrical stimulation	^[^ ^85^ ^]^
	Cell death	Fe‐doped LiNbO_3_ substrate	Light‐mediated electrical stimulation	^[^ ^83^ ^]^
		BiFeO_3_ nanoparticles	SHG	^[^ ^118^, ^119^ ^]^
	Bacterial killing	LiNbO_3_, LiTaO_3_ nanoparticles	Pyroelectrocatalytic generation of ROS	^[^ ^69d^ ^]^
		tetragonal‐BaTiO_3_ particles	Piezoelectrocatalytic generation of ROS	^[^ ^120^ ^]^
	Artificial retina	P(8‐AZO‐10)/P(VDF‐TrFE) composite membrane	Light‐mediated piezoelectrical stimulation	^[^ ^84^ ^]^
	Sensing cellular mechanics	PZT nanoribbons	Piezoelectric effect	^[^ ^94^ ^]^
	Cell‐line characterization	PVDF film	Infrared‐induced pyroelectric effect	^[^ ^95a^ ^]^
	Cell manipulation (trapping; separation; lysis; gene transfection)	LiNbO_3_ substrate	Inverse‐piezoelectric effect	^[^ ^32^, ^99^, ^102^, ^103^, ^104^ ^]^
	Cell labeling	LiNbO_3_, BaTiO_3_, KNbO_3_, and BiFeO_3_ nanoparticles	SHG	^[^ ^31^, ^32^, ^35^ ^]^
	Tooth whitening	BTO nanoparticles	Piezoelectrocatalytic generation of ROS	^[^ ^70^ ^]^
	Tumor eradication	BTO nanoparticles	Piezoelectrocatalytic generation of ROS	^[^ ^71^ ^]^
Tissue level	Bone tissue repair	BTO/P(VDF‐TrFE) composite film	Spontaneous polarization induced surface charge	^[^ ^65^ ^]^
		P(VDF‐TrFE)		
	Peripheral nerve regeneration	PVDF‐based scaffolds	Spontaneous polarization induced surface charge	^[^ ^67^ ^]^
	Recovery of Degenerative Dopaminergic Neurons	BaTiO3 nanoparticles with carbon shell	Piezoelectric generation of electromagnetic fields	^[^ ^79^ ^]^
	Remote‐manipulative nerve stimulation	Graphene sandwiched PVDF film	Pyroelectrical stimulation	^[^ ^92^ ^]^
	Precise sub‐organ neuromodulation	PZT ceramic array	Inverse‐piezoelectric mechanical stimulation	^[^ ^109^ ^]^
	Cardiac pacemakers	Pb(Mg_1/3_Nb_2/3_)O_3_‐PbTiO_3_ film	Piezoelectrical stimulation	^[^ ^71^, ^90^ ^]^
	Deep brain stimulation	Pb(In_1/2_Nb_1/2_)O_3_—Pb(Mg_1/3_Nb_2/3_)O_3_—PbTiO_3_ film	Piezoelectrical stimulation	^[^ ^91^ ^]^
	E‐skin	PVDF/ graphene microstructures	Piezoelectric, pyroelectric, and piezoresistive effect	^[^ ^97a^ ^]^
		Au/P3HT/P(VDF‐TrFE) with a PEDOT:PSS gate electrodes	Ferroelectric‐gate field‐effect	^[^ ^97c^ ^]^
	Cardiovascular disease diagnosis	PVDF composite nanofibers	Piezoelectric effect	^[^ ^98^ ^]^
	Bioimaging	BTO/KTiOPO_4_ nanoparticles, triphenylalanine peptide nanoassemblies	SHG	^[^ ^56^, ^113^, ^117^ ^]^
		Sr_3_Sn_2_O_7_:Nd^3+^ crystal	Piezoluminescence	^[^ ^114^ ^]^

Second, the use of FEMs for most bio‐related studies are in the preliminary stage and the relevant mechanisms underlining FEMs‐mediated biological intervention need to be further explored, such as the understanding of the cellular response when exposed to charged surfaces or the mechanism of mechanotransduction. In physics, ferroelectricity forms the basis of the random‐access memory and pyroelectricity has been widely for infrared sensors, which are relatively rare in biological materials. The understanding of ferroelectricity in physiological conditions, that is, bioferroelectricity, is not clear yet. Therefore, FEMs are not able to make a direct interaction with bioferroelectricity so far. However, by understanding bioferroelectricity the development of FEMs could make a great leap forward in the field of biomedicine for sure.

Third, biomedical applications of FEMs undergo unbalanced development in different fields. For example, ultrasound‐based devices have already been one of the most common setups for medical diagnoses and therapy, which are so commonly seen that people even ignore the existence of FEMs, the very key component hidden inside. In the field of tissue engineering, on the other hand, the utilization of FEMs demonstrating huge potential for repairing certain tissue defects via building up or restoring the bioelectrical microenvironment, is still in its infancy. Particularly for nonlinear optics, considerable number of harmonic labelers has been studied in the last few decades. There remains one challenging issue that harmonic labels will get dimmer when their size decreases, because the SHG intensity from the FEM crystals decreases accordingly with the square of the crystal volume. Considering the importance of precise presentation of biomolecular targets with high resolution in vivo, this limitation may hinder further development of nano‐scaled FEMs for nonlinear bioimaging. In addition, despite the fact NIR irradiation hold huge potential for deep‐tissue therapy, especially in brain science and neurology, photoregulation of biological events (i.e., optogenetics) via FEMs‐mediated harmonic process remains unexplored.

**Figure 18:**
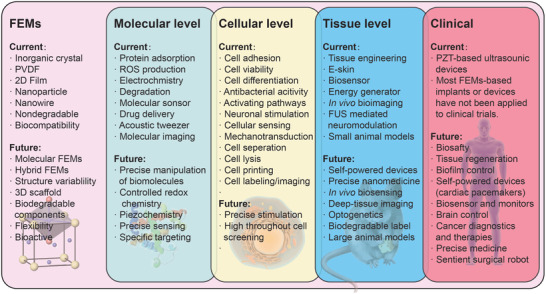
Summary of the current research developments and future perspectives related to FEMs and their biomedical applications.

In all, FEMs possess versatile properties and functionalities, making them one of the most intriguing biomaterials at present. From the material aspect, scientists can synthesize FEMs with novel features for complex needs. While from the biomedical aspect, FEMs already have demonstrated great potential to sensing or regulating biological events at different levels. Considering the bio‐application of FEMs is in its early stage with notable challenges ahead and many fields underexploited, there are reasons to have a great longing for a bright future of FEMs in the field of biomedicine (**Figure** **18**).

## Conflict of Interest

The authors declare no conflict of interest.
